# Clinical usefulness of a newly developed body surface navigation and monitoring system in radiotherapy

**DOI:** 10.1120/jacmp.v12i2.3400

**Published:** 2011-02-02

**Authors:** Hitoshi Takagi, Yasunori Obata, Hidetoshi Kobayashi, Kazuyuki Takenaka, Yasujirou Hirose, Hajime Goto, Tomohiko Hattori

**Affiliations:** ^1^ Nagoya University Postgraduate School of Health Sciences Nagoya Japan; ^2^ Ogaki Municipal Hospital Department of Radio technology Ogaki‐city Gifu Japan; ^3^ Nagoya University School of Health Sciences Nagoya Japan; ^4^ Fujita Health University School of Medicine Fujita Japan; ^5^ Nagoya City University Hospital Nagoya Japan; ^6^ Chubu Medical Co., Ltd. Yokkaichi‐city Mie Japan; ^7^ Sea phone Co., Ltd. Ogaki‐city Gifu Japan

**Keywords:** monitoring, positioning, body surface, contour

## Abstract

In radiotherapy, setup precision has great influence on the therapeutic effect. In addition, body movements during the irradiation and physical alternations during the treatment period might cause deviation from the planned irradiation dosage distribution. Both of these factors could undesirably influence the dose absorbed by the target. In order to solve these problems, we developed the “body surface navigation and monitoring system” (hereafter referred to as “Navi‐system”). The purpose of this study is to review the precision of the Navi‐system as well as its usefulness in clinical radiotherapy. The Navi‐system consists of a LED projector, a CCD camera, and a personal computer (PC). The LED projector projects 19 stripes on the patient's body and the CCD camera captures these stripes. The processed image of these stripes in color can be displayed on the PC monitor along with the patient's body surface image, and the digitalized results can be also displayed on the same monitor. The Navi‐system calculates the height of the body contour and the transverse height centroid for the 19 levels and compares them with the reference data to display the results on the monitor on a real‐time basis. These results are always replaced with new data after they are used for display; so, if the results need to be recorded, such recording commands should be given to the computer. 1) Evaluating the accuracy of the body surface height measurement: from the relationship between actual height changes and calculated height changes with torso surface by the Navi‐system, for the height changes from 0.0 mm to ± 10.0 mm, the changes show the underestimation of 1.0–1.5 mm and for ±11.0 mm to ± 20.0 mm, the underestimation of 1.5–3.0 mm. 2) Evaluating the accuracy of the transverse height centroid measurement: displacement of the inclined flat panel to the right by 5.0 mm, 10.0 mm, 15.0 mm and 20.0 mm showed the transverse height centroid calculated by the Navi‐system for 0.024±0.007 line/pair (mean ± SD), 0.045±0.006 line/pair, 0.066±0.006 line/pair and 0.089±0.007 line/pair, respectively. Also, displacement of the inclined flat panel to the left by 5.0 mm, 10.0 mm, 15.0 mm and 20.0 mm showed the transverse height centroid calculated by the Navi‐system for 0.015±0.007 line/pair (mean ± SD), 0.034±0.007 line/pair, 0.053±0.008 line/pair and 0.071±0.007 line/pair, respectively. 3) Clinical usefulness of the Navi‐system: on using the Navi‐system, the frequency of radiotherapy replanning increased from 5.2% to 21.8%, especially in pelvic or abdominal irradiation. We developed a new navigation system for the purpose of compensating for the weakness of MVCT, CBCT and other systems, as well as for having a screening function. This Navi‐system can monitor the patient continuously and measure change in height of the patient's body surface from the basic plane, in real time. It can also show the results both qualitatively and quantitatively on the PC monitor.

PACS number:87.52.‐g

## I. INTRODUCTION

The setup error may be reduced by using image‐guided radiation therapy (IGRT).^(^ ^1^ ^–^ ^5^ ^)^ In IGRT, there are several methods for obtaining the images and matching them to the references: megavoltage computed tomography (MVCT)^(6)^ and cone‐beam computed tomography (CBCT),^(^ ^7^ ^–^ ^10^ ^)^ in which an image of a target area is obtained directly and positioning is achieved by using the images of the target and the surrounding internal organs; linac‐graphs (LG) and electric portal imaging device (EPID), in which positioning is achieved by matching with an X‐ray image of the bone structure; Exac‐Trac system (ETS), in which the marker position is obtained by using an X‐ray and infrared camera; photogrammetry surface imaging system (PSIS),^(^ ^11^ ^–^ ^19^ ^)^ in which positioning is achieved by capturing and matching body surface contour images.

CBCT and MVCT, which are three dimensional matching systems, are considered to have optimum accuracy in acquiring the position of the target. However, except for the PSIS, those systems that are the same as the LG and the EPID cannot monitor patient movements during irradiation. Also, those systems cannot obtain the changes in the body contour (for example, due to weight loss or gain). Therefore, it is necessary for those systems to be used together with the PSIS.

We have developed a surface navigation and monitoring system, the Navi‐system, which can monitor body movements during irradiation, as well as changes in body contour, throughout the treatment period. If there is considerable change in the body contour, the radiotherapy treatment planning needs to be changed because the absorbed dose varies depending on the thickness of the patient's body. The developed system can aid setups on the couch by making comparisons with previous images. The purpose of this study is to evaluate the accuracy of this new Navi‐system and the clinical usefulness of monitoring body movements and changes in the body contour.

## II. MATERIALS AND METHODS

**Figure 1:**
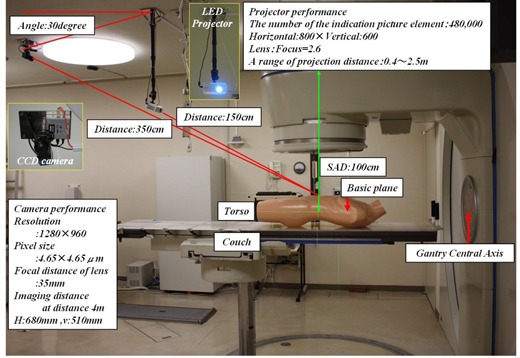
Disposition of Navi‐system in radiotherapy room.

Figure 1 shows the outline of our newly developed the Navi‐system. The Navi‐system consists of a LED projector (TDP FF1AJ, Toshiba Japan Co., Ltd.), a CCD camera (DFK 41AF02 FC, The Imaging Source Taiwan Co., Ltd.), and a personal computer (PC) (DIMENSION 9200, DELL Co., Ltd.). The LED projector projects 19 stripes on the patient's body, and the CCD camera captures these stripes. The processed image of these stripes in color can be displayed on the PC monitor with the patient's body surface image, and the digitalized results can also be displayed on the same monitor. In clinical treatment, an initial setup with the 3D laser pointer is performed as usual and the Navi‐system is used after that. It is not necessary to transfer the planning data from the radiation therapy planning system. The reference data are created at the first setup, and corrections will be made when differences from the reference data are detected at the subsequent setup. If the differences are very large, the treatment planning will need to be changed. After the setup, the Navi‐system is also used to check the setup precision at all times during the irradiation.

**Figure 2:**
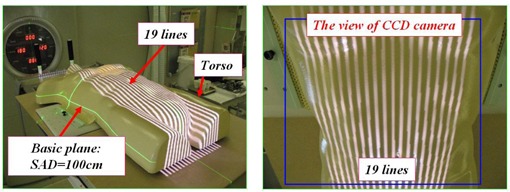
Nineteen lines projected on the torso, and the view of CCD camera.

The 19 stripes from the LED projector are adjusted with a trapezoidal correction over the large field including the irradiation field. Figure 2 shows an actual image of 19 stripes projected on a torso. The stripe width can be chosen from two types of width settings depending on the body width.

The CCD camera is fixed on the ceiling, away from the LED projector, at an angle of approximately 30° to the stripes. Figure 1 shows distances from the center of the basic plane. The CCD camera captures the 19 stripes in the effective visual field. The effective visual field of the CCD camera can be selected from three options (upper, middle or lower) in accordance with the irradiation field.

In order to evaluate the error in the setup by calculating the height of the body surface and the distortion or movement to the side, each of the 19 stripes is divided into 19 segments with an equal interval after they are captured by the CCD camera in the effective visual field. The basic plane is defined to be 100 cm away from the target for each segment, and the height from the basic plane is calculated. In order to calculate the height from the basic plane by using 2D images for a preliminary calibration, a flat panel is placed on the treatment couch, and the couch is moved from 0 mm (the basic plane) to 120 mm in increments of 10 mm. A matrix is then created to show the relation between pixel (horizontal on the monitor) and height (from the basic plane) for the centerpoint of each of the 19 segments of the 19 stripes. This system does not measure the height if it is beyond the basic plane.

In order to detect the difference between the current setup and the initial setup, the reference data about the body surface of the patient should be acquired initially when setups are done. The body surface has vertical motions associated with breathing, and a normal adult has a breathing cycle of approximately four to six seconds. This system therefore collects the data of body surface height continuously for 10 seconds with an interval of 0.11 seconds during any randomly selected period where the breathing cycle is stable. A typical breathing cycle is then calculated to decide the body surface height by using the calculation results. For each segment (19 by 19), the height is calculated, and a typical time is acquired by using the average of all the heights.

The average height can be obtained by Eq. (1):

(1) $$hm(t)=\sum_{n=1}^{19}\sum_{m=1}^{19}h(t,n,m)/(19\times 19)$$

where *hm(t)* is the average height of the body surface of all the segments (19 by 19) at the time t; *n* refers individually to the 19 stripes: n=1,2,3,…19 from the left to the right on looking from the foot on the table; *m* indicates the level in the transverse direction: m=1,2,3,…19 from the head to the foot; and *h(t, n, m)* is the height of each segment at the time *t*.

The typical time (T) is obtained by Eq. (2):

(2) $$hm(T)=1/\tau \int_{0}^{\tau }hm(t)dt$$

Because *hm(t)* are discrete data acquired every 0.11 seconds, they should be corrected for time *t* by using Bezier curves. In order to determine the typical time T, one breathing cycle τ is calculated as needed and T, where the average of hm(t) is consistent with hm(T), is determined. T can be obtained as two solutions for expiration and for inspiration; the former is used here for the typical time T. The height data of the body surface for 19 by 19 segments are acquired at T. Even if all data were acquired, it is impossible to revise the height of all of them. The degree of leaning of the body can be regulated if the degree of leaning of the craniocaudal direction can be grasped, and if a change of cross‐sectional height is provided, it is possible to account for the degree of the leaning during setup by the turn of the cross direction. Therefore, the reference height of a level is determined by taking the average of the data of the 19 segments in the transverse direction, and is determined by the Eq. (3):

(3) $$H(T,m)=\sum_{n=1}^{19}h(T,n,m)/19$$

In order to detect distortion or movement to the side, the transverse height centroid for the height of each level is calculated by Eq. (4):

(4) $$G(T,m)=\sum_{n=1}^{19}n\cdot \;\;h(T,n,m)/\sum_{n=1}^{19}h(T,n,m)$$

where *G(T, m)* is the transverse height centroid for the traversal height of a level *m* at the time *T*; and *h(T, n, m)* is the height of the stripe n for the level *m* at the time *T*. The transverse height centroid calculated at the first time should be the reference.

The Navi‐system always calculates the traversal height and the transverse height centroid for the 19 levels and compares them with the reference data to display the results on the monitor on a real‐time basis. These results are always replaced with new data after they have been used for the display; so, if the results need to be recorded, such recording commands should be given to the computer.

**Figure 3:**
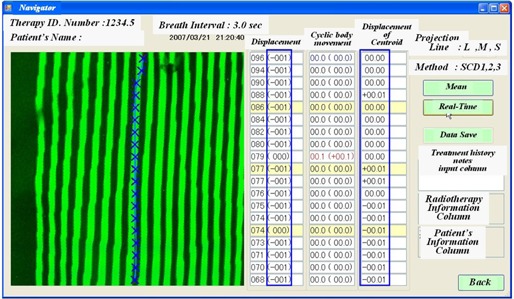
Information displayed on the monitor. A computed image of a patient's surface is shown on the left side. Next three columns show the height over the reference height (displacement), patient's cyclic body movement for ten seconds, and the displacement of the transverse height centroid.

Stripes of a patient's image at a typical time are processed in color by comparing the height data of the 19 levels (from head to foot) to the reference. The color is applied evenly to the 19 segments of the same level. Green indicates that the difference from the reference is 0 mm, while red indicates that the difference is larger than 10 mm. For the difference from 0 to +10 mm, the color changes from green to red in a phased manner. Blue indicates that the difference from the reference is smaller than −10 mm; for a difference ranging from 0 to 10 mm, the color changes from green to blue in a phased manner. As a qualitative result, the body surface image processed in color is displayed on the left half of the monitor (Fig. 3). As a quantitative result, the average heights of the 19 segments at the 19 levels and the differences (in mm) from the registered reference data are displayed on the left side of the right half on the monitor. The cyclic movements of the 19 levels on the body surface for the breathing period are displayed as standard deviations in the center of the right half of the monitor. The values within the parentheses are differences from the first registered reference data. Therefore, the maximum and minimum values in a breathing cycle can be acquired.

The transverse height centroid data as reference are displayed with 19 red X marks, and the transverse height centroid data as real‐time data (also as the saved data) are displayed with 19 blue X marks. Two kinds of X marks are severally connected, all with a line. As a quantitative result, the differences of the transverse height centroid from the reference are calculated and displayed on the right end of the monitor as a unit of line/pair. A movement to the left is displayed as a positive value, while that to the right is a negative value.

**Figure 4:**
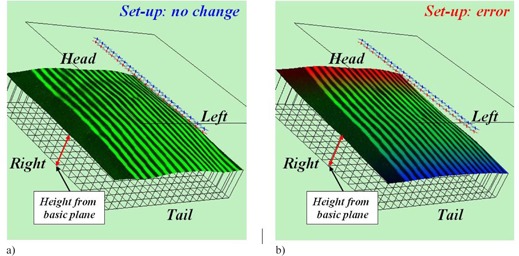
3‐D PC monitor images of Navi‐system: a) green area means no difference from the reference data; b) red and blue areas mean differences from the reference data;

In order to make visually clear a position on the body surface and the transverse height centroid, the display can be switched from normal to 3D as shown in Fig. 4, so that observation of the 3D body configuration from various angles becomes possible. If the heights of the patient's body surface are largely deviated from the first registered reference data, the color of the stripe will change automatically ((Figs. 4a), (b)). In the case that the transverse height centroid is largely deviated from the reference, the color will change to express the direction of the deviation.

### A. Evaluating the accuracy of the body surface height measurement

The precision in height measurement by the Navi‐system was determined by using a torso placed on a treatment couch and comparing the change in the height measured. More specifically, changes in height indicated by the ruler on the couch and changes indicated by the Navi‐system were compared. We set the basic plane (SAD: Source Axis Distance: 100.0 cm) at a depth of 7.0 cm from the surface of the torso. For the basic plane, we measured a change in the height of the torso surface in the field‐of‐vision domain of the Navi‐system by letting the height of the treatment couch increase in steps of 1.0 mm from −20.0 mm to +20.0 mm. In addition, we repeated these measurements three times to change to +20.0 mm in steps of 1.0 mm from −20.0 mm, and calculated the mean ± standard deviation of the height change of the 19 segments of the torso surface.

### B. Evaluating the accuracy of the transverse height centroid measurement

Displacement of the transverse height centroid was evaluated by moving an inclined flat panel on the couch. The panel, inclined at a 25° angle, was moved from 5.0 mm to 20.0 mm in steps of 5.0 mm and was monitored using the Navi‐system. Measurements were repeated 10 times for same movement, and the mean ± SD values were computed.

### C. Clinical usefulness of Navi‐system

The number of patients who underwent replanning were studied by comparing 938 patients without the Navi‐system (2004–2006) and 986 patients with the Navi‐system (2007–2009). With the Navi‐system, replanning was performed by detecting 10.0% of decrease or increase in thickness of the body surface contour. Without the Navi‐system, replanning was performed after long periods of stopping treatment and/or disagreement on the light beam field and the skin markers. (This clinical trial was carried out with the approval of the ethics board of Nagoya University School of Medicine (approval number: 6‐305)).

## III. RESULTS

### A. Evaluating the accuracy of the body surface height measurement with torso

**Figure 5:**
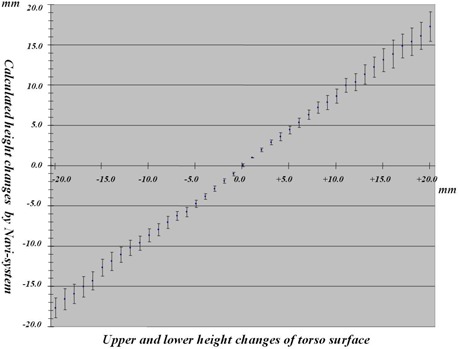
Relationship between actual and calculated height changes with torso.

Measurements of the average height change of the torso surface measured by the Navi‐system, in which the height of the treatment couch was increased in steps of 1.0 mm from −20.0 mm to +20.0 mm, are shown in Table 1. Fig. 5 shows the relationship between actual height changes and calculated height changes with torso surface by the Navi‐system. For height changes from 0.0 mm to ±10.0 mm, the underestimation is 1.0–1.5 mm, and, for ± 11.0 mm to ± 20.0 mm, the underestimation is 1.5–3.0 mm.

**Table 1:** Measurement of the average height change of the torso surface measured by the Navi‐system.

*Torso Height*	Calculated Height	Torso Height	*Calculated Height*
−20.0mm	−17.68±1.227 mm	1.0mm	1.00±0.00 mm
−19.0mm	−16.58±1.322 mm	2.0mm	1.95±0.225 mm
−18.0mm	−15.95±1.202 mm	3.0mm	2.89±0.310 mm
−17.0mm	−15.05±1.288 mm	4.0mm	3.58±0.498 mm
−16.0mm	−14.32±1.136 mm	5.0mm	4.42±0.498 mm
−15.0mm	−12.68±1.038 mm	6.0mm	5.32±0.572 mm
−14.0mm	−11.89±1.129 mm	7.0mm	6.32±0.572 mm
−13.0mm	−11.05±0.953 mm	8.0mm	7.21±0.700 mm
−12.0mm	−10.21±0.959 mm	9.0mm	7.84±0.882 mm
−11.0mm	−9.63±0.879 mm	10.0mm	8.63±0.879 mm
−10.0mm	−8.68±0.805 mm	11.0mm	9.95±0.853 mm
−9.0mm	−7.95±0.766 mm	12.0mm	10.37±1.046 mm
−8.0mm	−7.05±0.766 mm	13.0mm	11.32±1.183 mm
−7.0mm	−6.26±0.552 mm	14.0mm	12.21±1.250 mm
−6.0mm	−5.77±0.598 mm	15.0mm	13.11±1.423 mm
−5.0mm	−4.75±0.434 mm	16.0mm	13.84±1.473 mm
−4.0mm	−3.84±0.368 mm	17.0mm	14.84±1.473 mm
−3.0mm	−2.88±0.331 mm	18.0mm	15.37±1.707 mm
−2.0mm	−1.91±0.285 mm	19.0mm	16.11±1.666 mm
−1.0mm	−1.05±0.225 mm	20.0mm	17.26±1.818 mm
0.0mm	−0.04±0.186 mm		(mean±SD)

### B. Evaluating the accuracy of the transverse height centroid measurement

Displacement of the inclined flat panel to the right by 5.0 mm, 10.0 mm, 15.0 mm and 20.0 mm showed the transverse height centroid calculated by the Navi‐system for 0.024±0.007 line/pair (mean ± SD), 0.045±0.006 line/pair, 0.066±0.006 line/pair and 0.089±0.007 line/pair, respectively.

Displacement of the inclined flat panel to the left by 5.0 mm, 10.0 mm, 15.0 mm and 20.0 mm showed the transverse height centroid calculated by the Navi‐system for 0.015±0.007 line/pair, 0.034±0.007 line/pair, 0.053±0.008 line/pair and 0.071±0.007 line/pair, respectively.

Significant correlation occurred between the movement to the right or to the left of the inclined flat panel on the couch and the calculated center of the transverse height centroid by the Navi‐system (Table 2).

**Table 2:** Relationship between distance of movement to the right or to the left and calculated traverse height centroid points by Navi‐system.

*Distance of Movement to the Right from Center*	*Calculated Traverse Height Centroid by Navi‐system*
5.0 mm	0.024±0.007 line/pair
10.0 mm	0.045±0.006 line/pair
15.0 mm	0.066±0.006 line/pair
20.0 mm	0.089±0.007 line/pair (mean±SD)
*Distance of Movement to the Left from Center*	*Calculated Traverse Height Centroid by Navi‐system*
5.0 mm	0.015±0.007 line/pair
10.0 mm	0.034±0.007 line/pair
15.0 mm	0.053±0.008 line/pair
20.0 mm	0.071±0.007 line/pair (mean±SD)

### C. Clinical usefulness of Navi‐system

**Figure 6:**
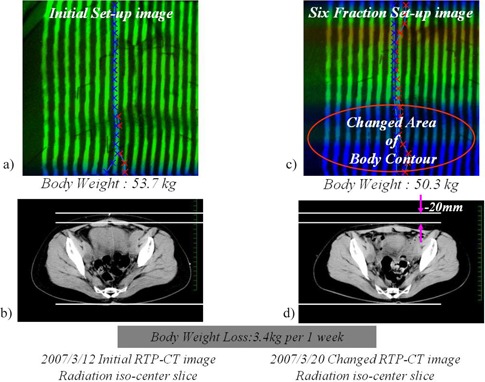
Clinical experience of Navi‐system: a) image at initial setup; b) CT scan at initial setup; c) image at the body weight loss of 3.4 kg; d) CT scan at the body weight loss of 3.4 kg.

Figure 6 shows changes in the body surface contour. This patient has lost 3.4 kg of body weight. The monitor shows a large decrease of the height of the body surface contour compared with the reference image. The CT scan image, which corresponds with these images, shows a 20.0 mm decrease in thickness (Fig.6).

**Figure 7:**
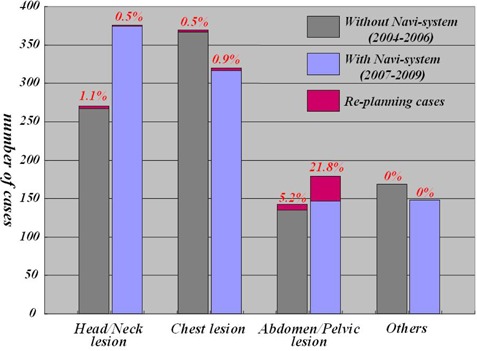
Comparison of replanning cases with and without Navi‐system.

The frequency of radiotherapy replanning increased from 5.2% to 21.8%, especially in pelvic or abdominal irradiation (Fig.7).

## IV. DISCUSSION

One of main issues in radiotherapy is improving the accuracy of the positioning setup. The accuracy of the setup has been frequently evaluated as the deviation of the center of the irradiated field from the previous skin mark.^(^ ^20^ ^–^ ^23^ ^)^ However, the patient's body surface is not flat but has a 3D surface on which irradiation fields are marked. Even if several points are fitted, the radiation field may not coincide with the planned target area. The Navi‐system can compare the surface field of the patient in real time by comparing the overall skin surface through mapping and color scaling. The Navi‐system has the benefit of accurate evaluation of the thickness of the patient's body throughout the radiotherapy period.

With the Navi‐system, only one CCD camera is required to evaluate the thickness of the patient's body. With most other systems, two cameras are required. Errors in a system with two cameras are larger than those in the Navi‐system. In addition the Navi‐system is cheaper than other systems because it uses a liquid crystal projector instead of a laser beam.

The surface image registration system can evaluate the accuracy of the setup error but cannot monitor movements during the irradiation because visualization is not in real time. The same problems are present in the 3D surface patient setup system,^(12)^ real‐time 3D surface image‐guided beam setup,^(13)^ optoelectronic sensing of body surface topology changes,^(14)^ and real‐time 3D motion analysis.^(15)^ A photogrammetry‐based patient positioning and monitoring system^(16)^ can monitor movements during irradiation but cannot evaluate changes in body contour. A stereo‐vision surface imaging system^(17)^ can monitor movements during irradiation but cannot differentiate between changes in the body contour and setup error. 3D optoelectronic analysis of interfractional patient setup variability^(18)^ uses markers attached to the surface of the patient's body and may have an error in detecting the positions of the markers. Moire analysis reported by Kobayashi et al.^(19)^ cannot compare the movements during irradiation. In the Navi‐system without the ionizing radiation beam, real‐time monitoring is possible without radiation exposure during irradiation from the beginning to the end. If the body movements of a patient during irradiation could be captured, we can suspend irradiation immediately to revise the setup in order to maintain irradiation accuracy.

When we evaluated calculated values by the Navi‐system by comparing with average height of actual measured values on a torso surface, there were errors of −1.0 to 1.5 mm for the height range 0.0 mm to ± 10.0 mm, and errors of −1.5 to 3.0 mm for the height range ± 11.00 mm to ± 20.0 mm. This was because the Navi‐system calculated the values as the difference between average height of a torso surface registered in the system as a reference data and a couch surface height. Thus, when the couch was moved, images captured by CCD camera were also displaced and caused the said errors. However, the greatest advantage of the Navi‐system is that it can detect this displacement intuitively. For this reason, we think the errors will not be a major problem.

Because the stripe width varies depending on the height from the basic plane, the calculated transverse height centroid is expressed in “line/pair” instead of the length in “mm”. We used the inclined flat panel to calculate the alternation of the transverse height centroid toward the sides and found the alternation was, at most, 0.1 line/pair. Though the values are small, it is easy to make qualitative judgments by the movement of the line consisting of X marks. If the panel is lying flat without a slope, there should be no alternation of the transverse height centroid. Clinically, the patient's body has a peak in the center and the height decreases toward the sides; the movement toward the sides is easy to detect by means of a change of the line.

By using the Navi‐system, 20.0% of the patients were found to need replanning for abdominal or pelvic radiotherapy. Without this replanning, most of the patients would have received a dosage higher than what was ultimately prescribed. The relationship between body weight loss and absorbed dosage was previously reported.^(^ ^24^ ^,^ ^25^ ^)^ Boda‐Heggemann et al.^(26)^ reported that stereotactic radiotherapy would not be affected by a change in the body contour because of the small fractional radiotherapy. On the other hand, by using the Navi‐system, it becomes possible to implement a revised treatment plan as required. It is preferable that the dose distribution within PTV should be consistent whenever possible, but it is permissible for the dose to be within the range of 95% to 107% of the reference dose.^(^ ^27^ ^,^ ^28^ ^)^ However, in reality, there are many cases existing outside the permissible range. In these cases, the acceptance of the dose distribution depends on the judgment and responsibility of the radiation oncologist in charge.^(^ ^29^ ^–^ ^34^ ^)^ By using the Navi‐system, such cases where the absorbed dose is outside the range of 95% to 107% of the reference dose can be extracted according to the quantitative result. For such cases, a revised treatment plan can be made and, by evaluating the difference of the dose reference point, the radiotherapy can be improved afterwards. It should be noted that the physical alternation is a threshold for variation with the change in the time. It should therefore be discussed further whether the criterion of “a revision of the treatment plan is required when the height from the basic plane changes by 10% or more” is appropriate or not.

## V. CONCLUSIONS

Radiotherapy devices with IGRT have become prevalent. We developed the Navi‐system to compensate for the weakness in systems like MVCT, CBCT and others, as well as to have a screening function. The Navi‐system can measure continuously (in real time) the height changes of the body surface, including the irradiation field, from the basic plane, as well as the transverse height centroid. In addition, those measurement results can be displayed both qualitatively and quantitatively on the PC monitor.

By using the Navi‐system, reproducibility can be improved by making minute adjustments to the setup on the treatment couch; body movements during treatment (after the setup until the start and the end of irradiation) can be monitored; and physical alternation during the radiotherapy period can be detected.

As a result, the Navi‐system can function as an assistive device to improve setup precision as well as irradiation accuracy in spite of body movements during irradiation. It can also play a role as an indicator of the decision criteria for the necessity of revision of the treatment plan to vary the reference dose based on physical alternations.

## ACKNOWLEDGMENTS

We would like to thank Ms. Toyoko Yanase and Ms. Takako Takahashi for their secretarial assistance.
